# Context-Aware Trust Prediction for Optimal Routing in Opportunistic IoT Systems

**DOI:** 10.3390/s25123672

**Published:** 2025-06-12

**Authors:** Abdulkadir Abdulahi Hasan, Xianwen Fang, Sohaib Latif, Adeel Iqbal

**Affiliations:** 1 School of Mathematics and Big Data, Anhui University of Science and Technology, Huainan 232001, China; engoscar8080@gmail.com; 2 Department of Computer Science and Software Engineering, Grand Asian University, Sialkot 51310, Pakistan; sohaib.latif@gaus.edu.pk; 3 School of Computer Science and Engineering, Yeungnam University, Gyeongsan-si 38541, Republic of Korea

**Keywords:** Social Opportunistic Internet of Things (SO-IoT), trust-based routing, intermediate node selection, social interest, reliability, Bayesian inference, Jeffrey’s conditioning, secure data dissemination, context-aware communication, single-hop routing, route optimization, trust-level computation, IoT security

## Abstract

The Social Opportunistic Internet of Things (SO-IoT) is a rapidly emerging paradigm that enables mobile, ad-hoc device communication based on both physical and social interactions. In such networks, routing decisions heavily depend on the selection of intermediate nodes to ensure secure and efficient data dissemination. Traditional approaches relying solely on reliability or social interest fail to capture the multifaceted trustworthiness of nodes in dynamic SO-IoT environments. This paper proposes a trust-based route optimization framework that integrates social interest and behavioral reliability using Bayesian inference and Jeffrey’s conditioning. A composite trust level is computed for each intermediate node to determine its suitability for data forwarding. To validate the framework, we conduct a two-phase simulation-based analysis: a scenario-driven evaluation that demonstrates the model’s behavior in controlled settings, and a large-scale NS-3-based simulation comparing our method with benchmark routing schemes, including random, greedy, and AI-based protocols. Results confirm that our proposed model achieves up to an 88.9% delivery ratio with minimal energy consumption and the highest trust accuracy (86.5%), demonstrating its robustness and scalability in real-world-inspired IoT environments.

## 1. Introduction

The rapid evolution of the Internet of Things (IoT) has enabled the seamless integration of physical devices into digital ecosystems, fostering intelligent automation [1] and ubiquitous connectivity [2]. These connected devices range from wearable health monitors [3] to autonomous vehicles [4], interacting over short-range [5] and long-range [6] wireless networks. With the expansion of IoT into more human-centric environments, the concept of the Social IoT (SIoT) has emerged, where devices interact not just based on physical proximity or protocol-level compatibility, but also through social relationships such as co-location, ownership, and shared interest [7]. A more dynamic subset of this paradigm, referred to as the Socially Oriented Internet of Things (SO-IoT) [8], emphasizes opportunistic, infrastructure-less interactions among mobile entities that exploit social cues for collaboration and routing, focuses on dynamic and decentralized environments where devices communicate opportunistically, without fixed infrastructure, and rely on intermediate nodes for data transmission. In these networks, intermediate nodes are not pre-established; instead, they are selected in real time based on context. Such spontaneity introduces challenges in ensuring secure and trustworthy routing [9].

Traditionally, routing in opportunistic networks has been tackled through protocols such as Routing Protocol for Low-Power and Lossy Networks (RPL) [10]. While effective in structured environments, RPL and similar protocols often fall short in dynamically changing social settings due to their lack of integrated trust management. This has prompted researchers to explore trust-based routing frameworks [11], where trustworthiness is derived from a node’s past behavior and contextual attributes [12]. Existing approaches tend to focus on either social affinity [13] (e.g., shared interests or group affiliations) or behavioral reliability [9] (e.g., successful message-forwarding history), rather than an integrated view of both. A node might share common interests but may act maliciously or unreliably, and vice versa. Therefore, a dual-dimensional trust framework is essential, one that explicitly distinguishes between social trust (derived from social compatibility and interest alignment) and behavioral reliability (based on historical message-forwarding performance), to enable robust and context-aware routing in SO-IoT systems.

**Figure 1:**
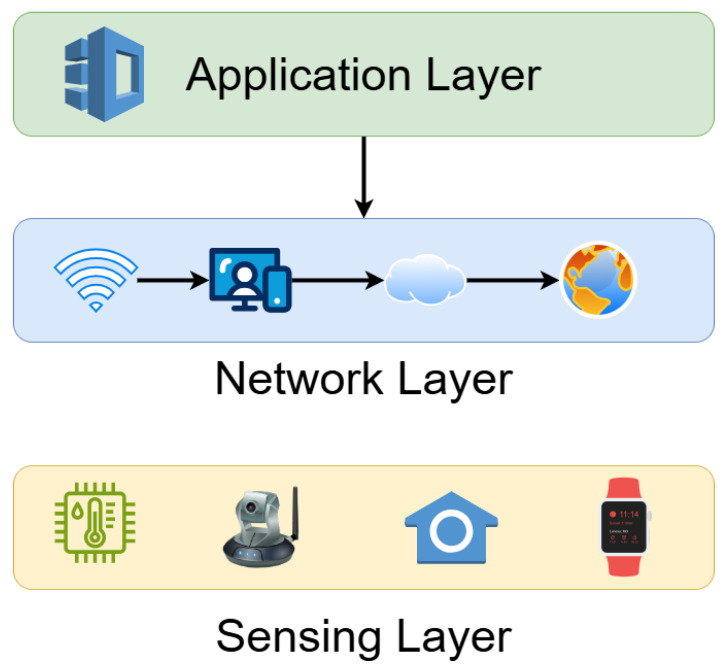
Layered architecture of a generic IoT system, highlighting the perception, network, and application layers. The trust-aware routing framework in this study operates at the network layer, focusing on secure and context-aware data forwarding.

This paper proposes a trust-aware route optimization framework that evaluates each potential intermediate node based on two primary components: social interest, modeled via beta-distribution probability [14]; and reliability, derived using Bayesian inference [15,16]. These two factors are combined using Jeffrey’s conditioning [17] to compute a dynamic and comprehensive trust level, which the sender node uses to make optimal forwarding decisions. Figure 1 illustrates the layered structure of IoT systems, underscoring the communication role of the network layer, which this work directly enhances through trust-based route optimization.

**Figure 2:**
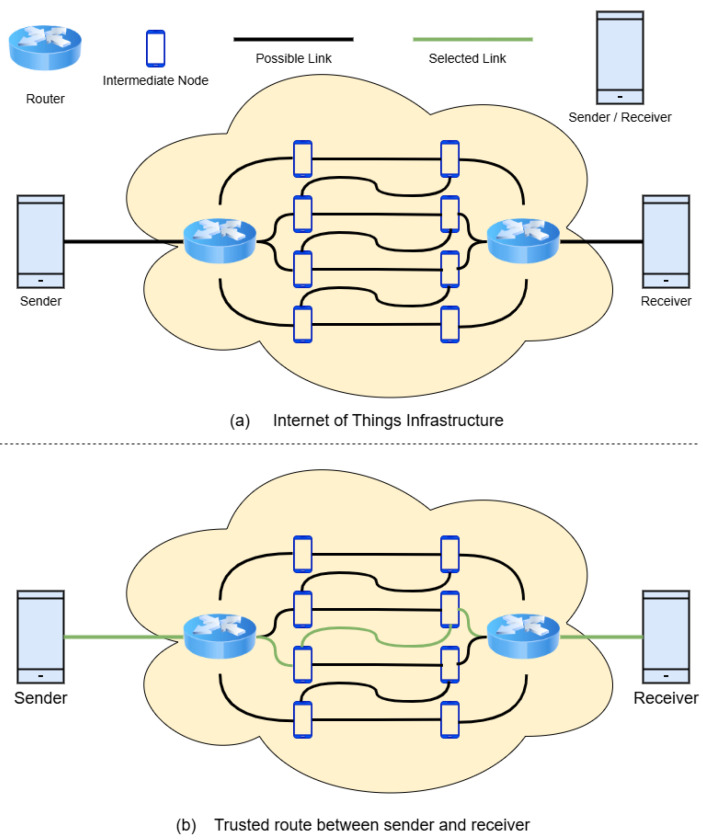
Illustration of the SO-IoT paradigm, where mobile IoT nodes opportunistically communicate via intermediate nodes without relying on fixed infrastructure. The figure highlights real-time trust-based decision-making for secure data forwarding.

The proposed approach is implemented in a single-hop communication setting, where the sender and receiver are connected through only one intermediate node, mirroring real-world scenarios such as mobile health monitoring or emergency response systems. Figure 2 depicts the SO-IoT paradigm, highlighting the unpredictability of links and the reliance on mobile, socially aware nodes.

Main Contributions:

- We propose a novel trust-aware routing framework for SO-IoT that integrates social compatibility and behavioral reliability into a unified trust-level computation model.
- A comprehensive trust evaluation mechanism is developed using Bayesian inference and Jeffrey’s conditioning to dynamically assess node behavior in uncertain environments.
- The proposed model is validated through scenario-based simulations using synthetic datasets, demonstrating its adaptability across varied network conditions.
- A large-scale NS-3 simulation is conducted to benchmark the proposed method against traditional and AI-based routing schemes, highlighting its superior performance in delivery ratio, latency, energy consumption, routing overhead, and trust accuracy.
- The results confirm the scalability, efficiency, and robustness of our approach, making it suitable for deployment in dynamic, resource-constrained SO-IoT systems.

The rest of this paper is organized as follows: Section 2 presents the related literature and discusses existing trust models and routing approaches. Section 3 describes the proposed system model and formal trust computation framework. Section 4 details the simulation setup and results. Section 5 concludes the paper and outlines directions for future research.

## 2. Related Work

Trust-based routing in SO-IoT has emerged as a vital strategy to improve data delivery in intermittently connected IoT networks. In SO-IoT (a blend of opportunistic networks and social IoT), devices rely on peer cooperation for store-carry-forward routing, but malicious or selfish nodes may refuse to forward packets, causing packet loss and delays [14]. Recent research has therefore focused on routing protocols that evaluate the trustworthiness of nodes based on past behavior or social relationships to route around uncooperative participants. We survey several trust-based routing protocols proposed in the last five years for SO-IoT and compare their core ideas and results. A few complementary trust-routing efforts in general IoT networks are also noted.

HIRouter [18] integrates node “intimacy” (social tie strength) and trust value into the forwarding decision. Nodes compute intimacy and trust from encounter histories, prioritizing more “intimate” and trustworthy neighbors for message relaying. This approach balances delivery efficiency and security. Simulations show that HIRouter significantly improves delivery rate and reduces overhead compared to baseline opportunistic routing.

SEER (Social relationship-based Energy-Efficient Routing) [19] incorporates social ties and resource awareness. Encounters are evaluated based on social bond strength, residual energy, and buffer capacity. SEER favors socially connected, resource-rich nodes, achieving higher delivery probability and lower overhead than epidemic and probabilistic schemes in simulations.

TBRP (Trust-Based Routing Protocol) [9] formulates relay selection as a multi-objective optimization problem solved using NSGA-II. It uses four-tier contextual trust metrics (e.g., encounter frequency, honesty, energy) to evolve optimal routes. TBRP achieved up to 58.57% higher delivery success than PRoPHET and notable gains over other trust-based schemes in simulations using real mobility traces.

CATR (Context-Aware Trust and Reputation Routing) [14] employs Bayesian trust modeling with a beta probability density function. It dynamically estimates trust based on contextual encounters and filters out malicious nodes. In simulations, CATR reduced average latency by 22%, dropped messages by 15%, and hop count by 9% compared to baselines.

BeRout [20] introduces benevolence-based trust, rewarding nodes that historically cooperate in forwarding. It includes a buffer management policy that prioritizes packets from trustworthy sources. Simulations confirm that BeRout improves delivery in resource-constrained settings by incentivizing cooperative behavior.

Recent studies also expand on social and contextual modeling in SO-IoT. SOScope (Social Object Scope) modeling [21] proposes a framework for computing the scope of social objects in Multi-IoT settings, emphasizing service discovery through object-level social contexts. TMSN-Survey (Temporal Mobile Social Network Survey) [22] surveys temporal behavior patterns in mobile social networks, which inform trust and routing decisions. ACRP (Adaptive Constraint-based Routing Protocol) [23] presents a multi-constraint adaptive routing protocol to maximize network lifetime, relevant to our goals of lightweight trust computation under constrained conditions.

Table 1 summarizes the key features of the above trust-based routing protocols in SO-IoT. Each scheme uniquely leverages trust or social context—whether through AI optimization, energy-aware routing, probabilistic models, or behavioral incentives—yet all report substantial improvements over classical routing strategies. These findings collectively highlight the critical role of trust in enhancing delivery reliability, resilience, and efficiency in opportunistic IoT environments.

Beyond opportunistic scenarios, trust-based routing is also explored in IoT. For instance, Muzammal et al. [24] propose SMTrust for securing RPL-based IoT routing against Rank and Blackhole attacks. Their framework uses mobility-aware trust metrics, improving throughput by 35% and reducing packet loss by 45%, with only marginal energy cost. The surge of trust-aware protocols illustrates that embedding social context and behavioral analysis into routing decisions is pivotal for scalable and secure IoT communication, particularly in decentralized and delay-tolerant scenarios.

## 3. System Model

In SO-IoT networks, trust prediction plays a pivotal role in optimizing route selection for reliable data dissemination. Given the transient and mobile nature of IoT devices, direct communication between sender and receiver is rarely feasible, requiring intermediate nodes. This section introduces a comprehensive system model that facilitates trusted intermediate node selection by integrating contextual information—namely, social interests and behavioral reliability—using Bayesian inference and Jeffrey’s conditioning.

**Figure 3:**
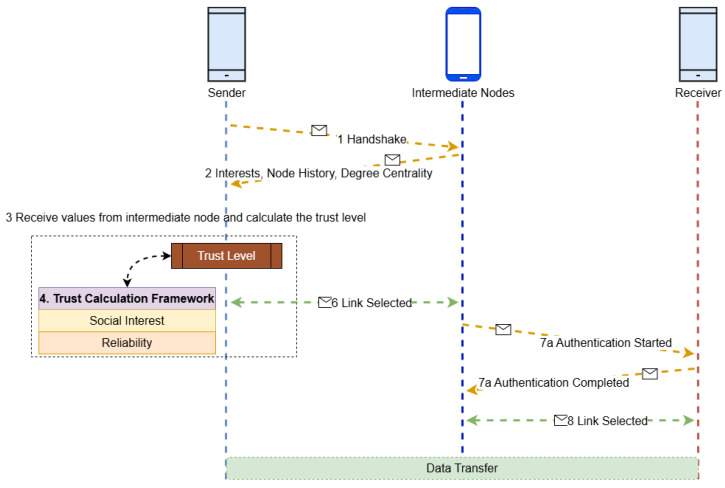
Conceptual architecture of trust-based node selection in a SO-IoT environment. The sender evaluates multiple candidate intermediate nodes based on social interest and behavioral reliability to select the most trustworthy path to the receiver.

The architecture, depicted in Figure 3, comprises a sender node, multiple candidate intermediate nodes, and the receiver node. The sender node evaluates potential intermediaries using historical logs and computed metrics to identify the most trusted node for forwarding data. Trust is not absolute but probabilistic, evolving as nodes accumulate interaction history.

The system model operates through the following stages:

- Input Acquisition: Each intermediate node maintains logs of its interactions, including successful and failed message exchanges and contextually similar service records.
- Social Compatibility Estimation: The semantic alignment of nodes is computed using a social interest model based on a beta distribution.
- Reliability Estimation: Behavioral consistency is derived from the node’s message-forwarding history using Bayesian methods.
- Trust Integration: Social compatibility and reliability are combined to compute a composite trust level.
- Optimal Node Selection: The node with the highest trust level is selected for secure data transfer.

Although the SO-IoT infrastructure supports multi-hop communication, our model focuses on a single-hop setup. This simplification allows detailed analysis of trust metrics between the sender and a single intermediate node before data reaches the destination. Multi-hop extension remains a subject for future work. In our proposed model, we formally define social trust as the probabilistic expectation of cooperation based on shared social context, modeled via interest alignment using a beta distribution. Behavioral reliability, on the other hand, quantifies how consistently a node has behaved according to expected service behavior, derived via Bayesian inference. These two orthogonal components are fused using a weighted approach to form the composite trust level, which directly informs the intermediate node selection process.

Each intermediate node is evaluated based on how well its social interests align with those of the sender. Interests are labeled as $I_{1},I_{2},\ldots ,I_{k}$, and a node *m* is assessed by node *n* using the following probability-based trust metric:

(1) $$T_{nm}^{I_{k}}={\left(P_{nm}^{I_{k}}\right)}^{{\left(1-P_{nm}^{I_{k}}\right)}^{y^{2}}}$$

where $P_{nm}^{I_{k}}$ is the probability that node *m* satisfies interest $I_{k}$ for node *n*, and $y^{2}$ is a severity parameter controlling the sensitivity of trust.

(2) $$P_{nm}^{I_{k}}=\frac{SR_{nm}^{I_{k}}+1}{SR_{nm}^{I_{k}}+UR_{nm}^{I_{k}}+2}$$

Here:

- $SR_{nm}^{I_{k}}$ = total successful requests related to $I_{k}$ from *n* to *m*;
- $UR_{nm}^{I_{k}}$ = total failed requests related to $I_{k}$.

The global direct social trust for node *m* is computed as:

(3) $$T_{nm}^{gd}=\sum_{I_{k}\in \Phi }\rho \omega_{I_{k}}\;\cdot \;T_{nm}^{I_{k}}$$

where $\rho \omega_{I_{k}}$ is the normalized weight for each interest.

Reliability reflects behavioral consistency. A node frequently denying requests or exhibiting unpredictable behavior will receive a lower reliability score. Using a Bayesian update rule, reliability $R_{m}$ of node *m* is given by:

(4) $$R_{m}=\frac{MF+1}{MF+MD+2}$$

where MF and MD are counts of message forwards and denials, respectively.

The trust level combines the semantic compatibility and reliability of the intermediate node:

(5) $$TL_{nm}=T_{nm}^{gd}+R_{m}$$

A higher trust level indicates a stronger candidate for data forwarding. Nodes are ranked accordingly.

To account for uncertainty and dynamically evolving node behavior, we apply Jeffrey’s conditioning to revise beliefs about node behavior based on partial evidence. The predictive trust in behavior θ is estimated using:

(6) $$P\left(\theta \right)=\beta (SR_{nm}^{I_{k}}\left(\Delta t\right)+1,UR_{nm}^{I_{k}}\left(\Delta t\right)+1)$$

where Δt defines the observation window.

Algorithm 1 ensures optimal route selection by dynamically evaluating the social and behavioral metrics of candidate intermediate nodes in SO-IoT networks.
**Algorithm 1** Enhanced Trust-Based Intermediate Node Selection  1:**Input:** Candidate node set N, interaction logs  2:**Output:** Node with the highest composite trust level  3:**for** each node m∈N **do**  4:    **for** each interest $I_{k}$ **do**  5:        Compute $P_{nm}^{I_{k}}=\frac{SR_{nm}^{I_{k}}+1}{SR_{nm}^{I_{k}}+UR_{nm}^{I_{k}}+2}$  6:        Compute $T_{nm}^{I_{k}}={\left(P_{nm}^{I_{k}}\right)}^{{\left(1-P_{nm}^{I_{k}}\right)}^{y^{2}}}$  7:    **end for**  8:    Compute $T_{nm}^{gd}=\sum \rho \omega_{I_{k}}\;\cdot \;T_{nm}^{I_{k}}$  9:    Compute reliability $R_{m}=\frac{MF+1}{MF+MD+2}$10:    Compute confidence $C_{nm}=\frac{SR_{nm}+UR_{nm}}{SR_{\max}+UR_{\max}}$11:    Compute trust level $TL_{nm}=C_{nm}\;\cdot \;\left(\alpha \;\cdot \;T_{nm}^{gd}+\left(1-\alpha \right)\;\cdot \;R_{m}\right)$12:**end for**13:**return** Node *m* with maximum $TL_{nm}$

### 3.1. Resilience to Adversarial Trust Manipulation

The proposed trust model offers partial resilience to adversarial behaviors such as Sybil attacks and collusion. Unlike models that rely on aggregated ratings, our approach emphasizes direct interactions and behavioral evidence, reducing the influence of fabricated trust scores from malicious nodes. However, we acknowledge that the current model does not fully address large-scale Sybil infiltration or coordinated collusion. To enhance security, future extensions will incorporate identity verification mechanisms (e.g., device fingerprinting or blockchain-backed identities), trust score decay models to detect sudden behavioral shifts, and anomaly detection using mini-batch supervised learning. These directions align with recent findings on DoS-resilient control systems and secure controller design using machine learning supervision [25,26].

### 3.2. Toward Multi-Hop Trust Routing

While this study focuses on single-hop trust evaluation, the proposed model is amenable to multi-hop extensions. In such cases, each node would aggregate trust scores not only from direct neighbors but also via trust propagation from downstream nodes. To mitigate misinformation, trust decay functions and credibility filters would be applied to reduce the influence of less reliable indirect paths. Route selection could then consider both composite trust and end-to-end QoS metrics (e.g., delay, hop count). Additionally, inspired by recent work on event-triggered control with mini-batch machine learning [25] and compressed control in fuzzy NCSS under DoS attacks [27], we propose future integration of lightweight learning modules at edge nodes. These could dynamically adjust trust thresholds and improve resilience in adversarial or large-scale multi-hop deployments.

## 4. Simulation and Results

The emergence of SO-IoT has led to a growing demand for reliable, context-aware routing mechanisms to cope with dynamic node behavior and heterogeneous trustworthiness. This section presents a two-part simulation study of the proposed trust-aware node selection framework. The first part involves scenario-based evaluations using synthetically generated datasets to analyze trust behavior in controlled topologies. The second part benchmarks the proposed method against conventional and AI-based routing protocols using a real-world scale NS-3 simulation environment. Together, these complementary analyses assess the method’s performance under both illustrative and realistic conditions.

### 4.1. Scenario-Based Evaluation

This section presents a detailed simulation study evaluating the proposed trust-aware node selection strategy using synthetically generated datasets. Three different scenarios of increasing network complexity are examined, and performance comparisons are made against traditional selection techniques relying solely on either social interest or reliability. A dataset comprising 500 IoT nodes was synthetically generated using MS Excel version 1808, later converted into a .csv format for computational modeling. Python 3.11 was used for simulations, leveraging the pandas 2.3.0 library to load and manipulate data within Visual Studio 2022 Code. Trust metrics, specifically social interest, reliability, and composite trust level, were computed for each intermediate node based on the trust computation framework outlined in Section 3. The topology of SO-IoT networks and their routing paths were visualized using the Gephi 0.10.1 tool, offering insights into connectivity and trust propagation.

**Figure 4:**
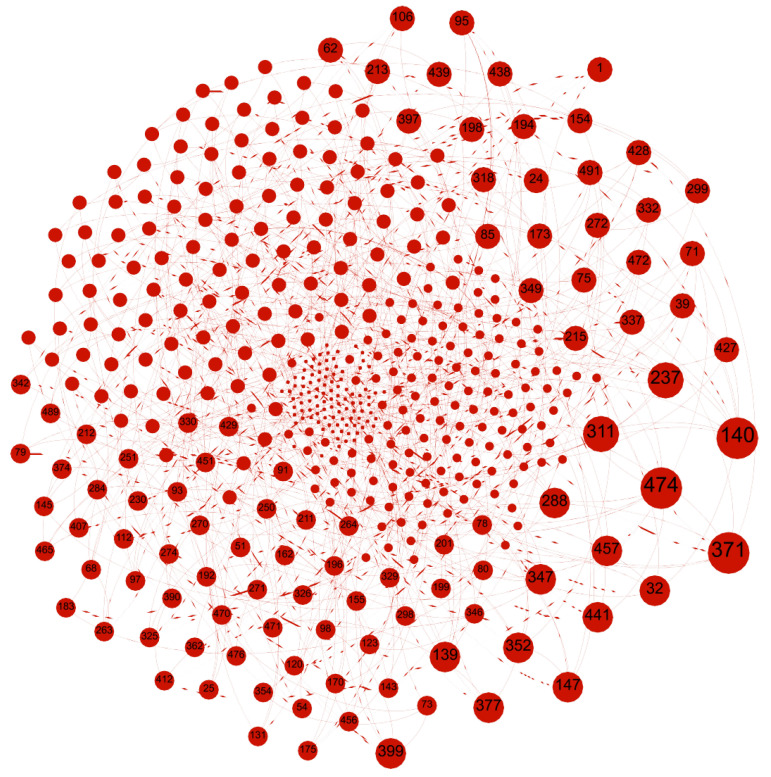
Visualized topology of a SO-IoT network using Gephi, depicting mobile nodes with no fixed communication path. Nodes dynamically form links based on social and trust parameters to forward data securely.

Figure 4 illustrates the SO-IoT paradigm, which is visualized using Gephi. The evaluation is conducted using a single-hop model, focusing on a single intermediate node selection for data forwarding between the sender and receiver. Three distinct SO-IoT scenarios were simulated to validate the robustness and effectiveness of the proposed trust-based routing framework. These scenarios differ in terms of network size and intermediate node density, allowing a comprehensive analysis of trust-level computation and route optimization. This section presents the evaluation of Scenario 1 in detail.

#### 4.1.1. Scenario 1: Trust Evaluation with Five Intermediate Nodes

**Figure 5:**
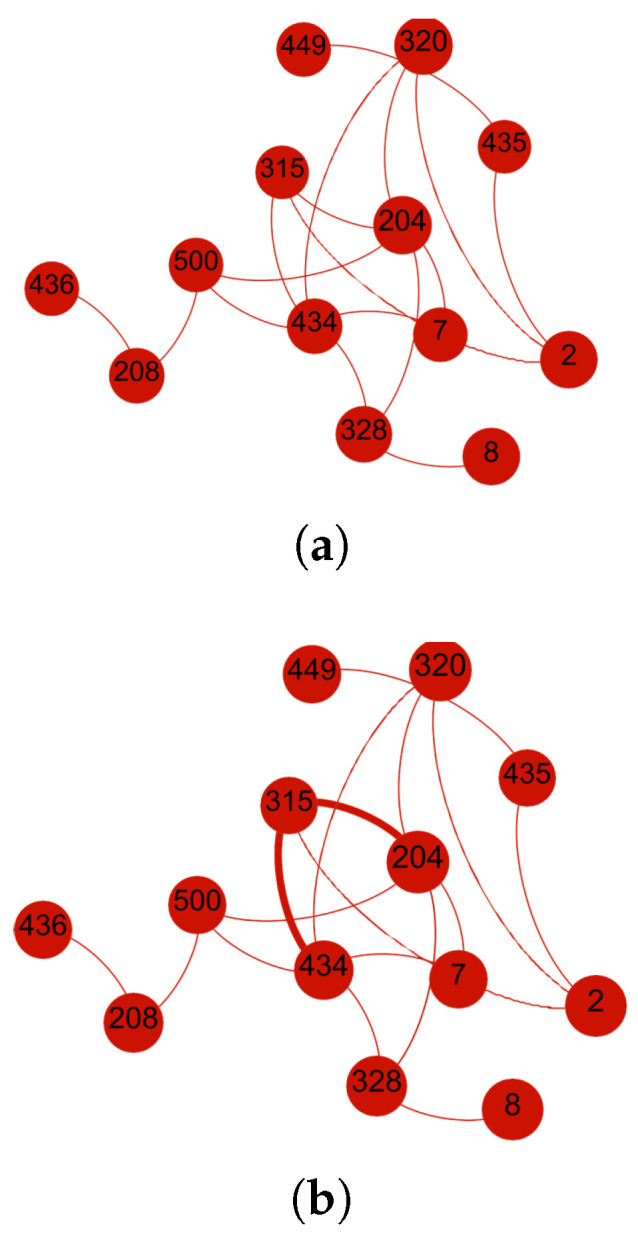
Scenario 1: The sender evaluates five intermediate nodes based on computed trust metrics. The selected route maximizes composite trust, balancing social interest and behavioral reliability. (**a**) Initial network layout with sender Node 434, receiver Node 204, and five intermediate candidates. (**b**) Final communication path established through Node 315, selected based on the highest composite trust level.

In this scenario, the SO-IoT network comprises thirteen nodes, where Node 434 acts as the sender and Node 204 as the receiver. The sender and receiver do not have a direct communication link and must rely on one of the five available intermediate nodes to forward the data. The network topology is illustrated in Figure 5a.

Upon initiating communication, the sender broadcasts a handshake packet. Intermediate nodes willing to participate respond with their trust-relevant parameters, including social interest metrics, message forwarding history, and availability. Using this information, the sender computes three key metrics: social interest, reliability, and the final trust level for each node.

**Figure 6:**
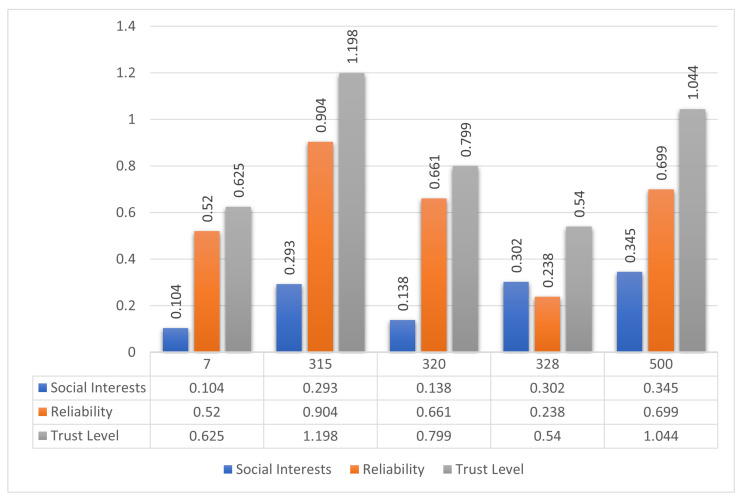
Comparison of social interest, behavioral reliability, and composite trust levels for five candidate nodes in Scenario 1. Node 315 is selected for data forwarding due to its highest overall trust score.

The computed values are shown in Figure 6. Among the nodes, Node 500 demonstrates the highest social interest, while Node 315 achieves the highest reliability. However, when combining these metrics through the proposed trust model, Node 315 yields the highest overall trust level and is thus selected as the optimal forwarding node.

Once trust levels are computed, the sender prioritizes the intermediate nodes based on their scores and selects Node 315 to forward the data to the receiver. Figure 5b shows the optimized trust-based communication route between sender and receiver.

**Figure 7:**
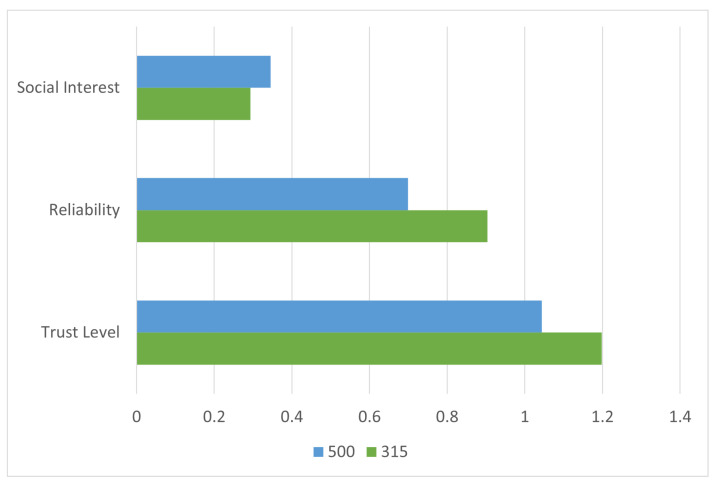
Node selection comparison in Scenario 1 based on three criteria: social interest, reliability, and composite trust. While Node 500 ranks highest in interest and Node 315 in reliability, the trust model selects Node 315 as the optimal forwarder.

To evaluate the comparative performance of the proposed method, a baseline analysis is conducted using conventional node selection strategies based solely on either social interest or reliability. Figure 7 illustrates that although Node 500 ranks highest in social interest and Node 315 in reliability, the proposed model’s combination of both yields a superior decision in selecting Node 315.

This scenario validates the capability of the proposed trust-based routing model to effectively integrate multiple trust dimensions and outperform traditional techniques that rely on singular metrics. The model enhances route reliability, trustworthiness, and communication security in opportunistic IoT environments.

#### 4.1.2. Scenario 2: Trust Evaluation with Six Intermediate Nodes

**Figure 8:**
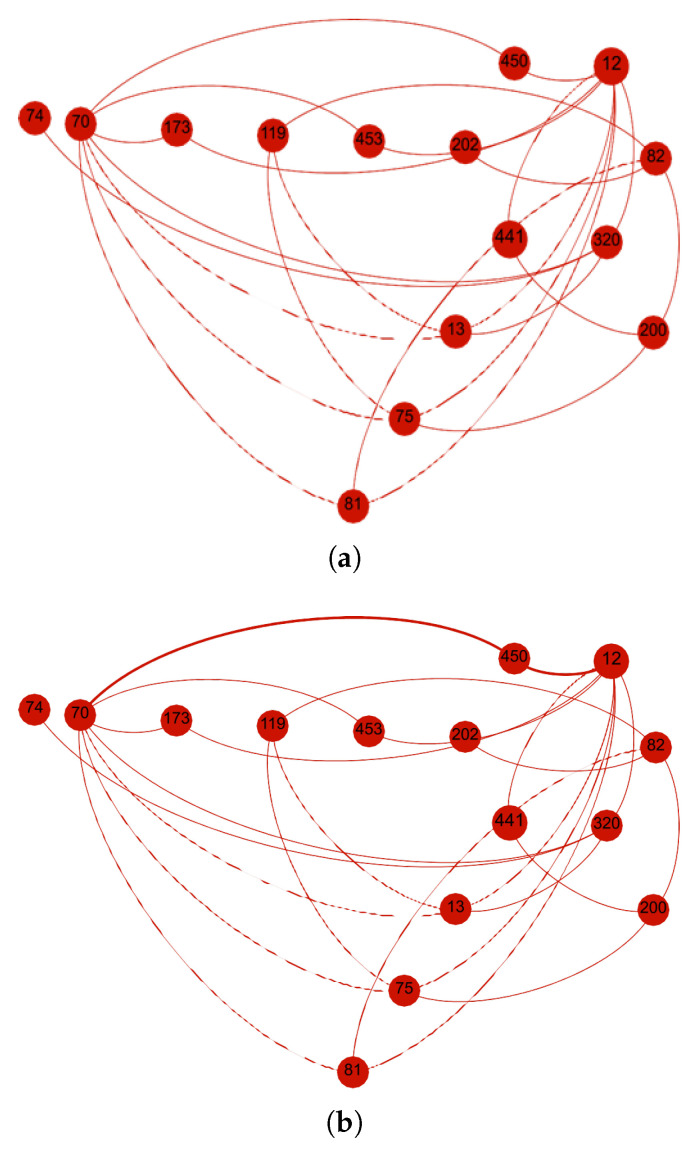
Scenario 2: Trust metrics are computed for six intermediate nodes, enabling the sender to select the most reliable and socially aligned node for data forwarding. (**a**) Network topology with sender Node 12, receiver Node 70, and six forwarding candidates. (**b**) Route established through Node 450 based on the highest composite trust.

In the second scenario, the Social Opportunistic IoT (SO-IoT) network comprises fifteen nodes, including six intermediate nodes that can potentially forward data. Node 12 is designated as the sender, while Node 70 acts as the receiver. Due to the lack of a direct communication path between them, the sender must evaluate available intermediate nodes to establish a trusted single-hop route. The network structure for this scenario is shown in Figure 8a.

Following the initial handshake process, all intermediate nodes respond with their corresponding trust-relevant attributes, including social interest vectors, message delivery history, and availability metrics. The sender computes each node’s social interest, reliability, and composite trust level by applying the trust calculation framework.

**Figure 9:**
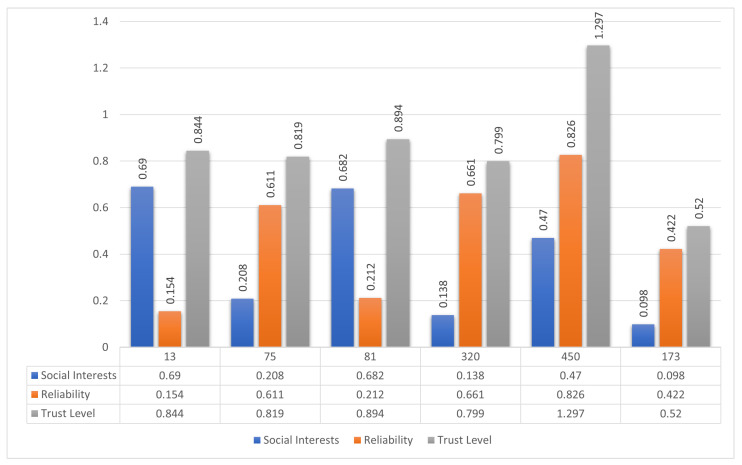
Social interest, reliability, and trust-level comparison for six intermediate nodes in Scenario 2. Node 450 achieves the highest composite trust and is selected for message forwarding.

Figure 9 presents a graphical representation of the calculated trust values. While Node 13 shows the highest social interest score, and Node 450 leads in reliability, the integrated trust evaluation identifies Node 450 as having the highest overall trust level. Thus, Node 450 is selected as the most reliable and trustworthy forwarding candidate.

Upon determining trust levels, the sender prioritizes Node 450 for routing. As shown in Figure 8b, the sender initiates data transfer through the selected intermediate node. This trust-aware route minimizes the risk of data loss or manipulation by ensuring that only the most qualified intermediate node handles the forwarding task.

**Figure 10:**
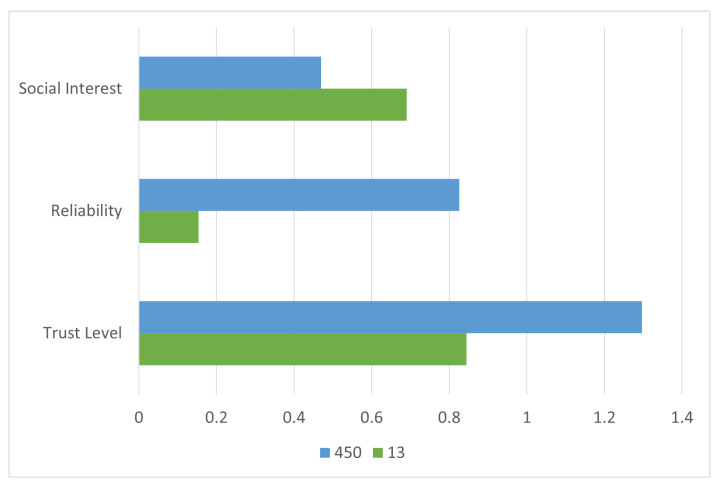
Comparison of node selection in Scenario 2 using individual metrics versus the proposed composite trust model. The unified trust score provides more robust and context-aware node evaluation.

To assess the performance of the proposed model against traditional methods, the node selection results are compared with those based solely on either social interest or reliability. As illustrated in Figure 10, while Node 13 performs best in terms of social compatibility, and Node 450 in reliability, only the composite trust model is able to accurately identify Node 450 as the optimal forwarding agent. This demonstrates the added value of multi-dimensional trust assessment in SO-IoT.

The outcome of this scenario highlights the importance of combining both social and behavioral trust indicators. The proposed framework proves effective in identifying the most suitable intermediate node, offering improved reliability and security compared to conventional selection strategies.

#### 4.1.3. Scenario 3: Trust Evaluation with Nine Intermediate Nodes

**Figure 11:**
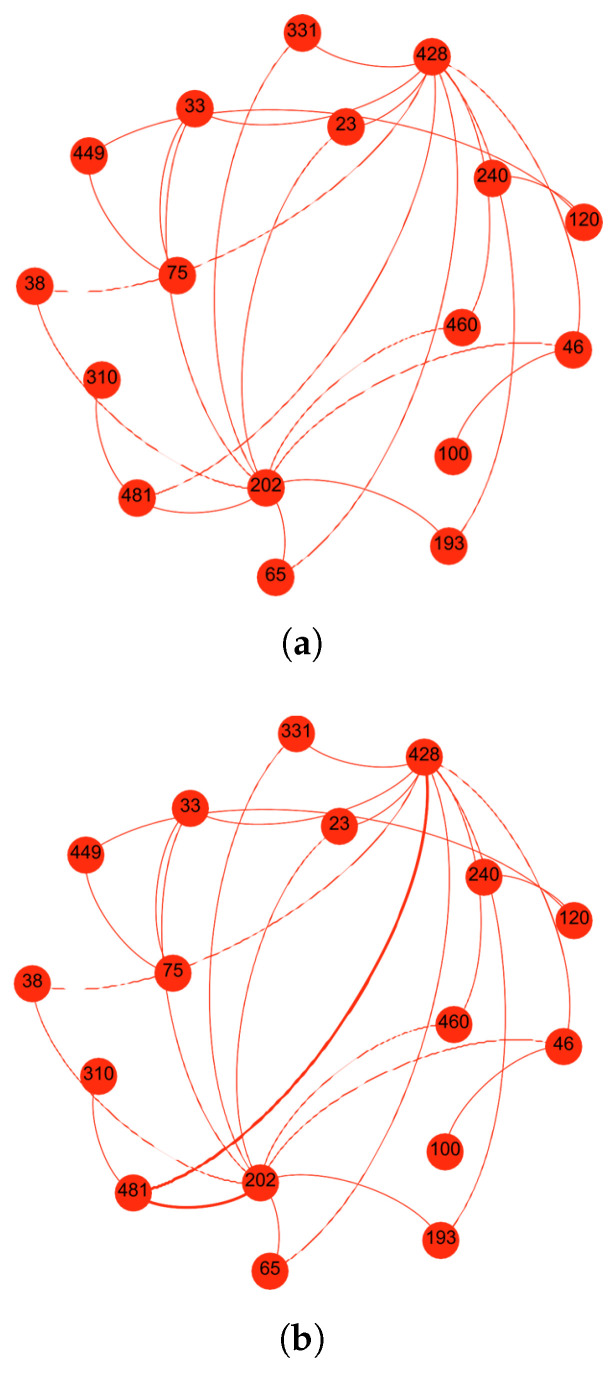
Scenario 3: Among nine available intermediaries, the proposed framework identifies the optimal forwarding node by integrating social and behavioral trust indicators, ensuring secure and context-aware communication. (**a**) Network topology with sender Node 428, receiver Node 202, and nine intermediate candidates. (**b**) Route formed through Node 481, identified as the most trusted intermediary.

The third scenario examines a more complex SO-IoT network consisting of seventeen nodes. Node 428 is designated as the sender, while Node 202 is the intended receiver. Since no direct communication link exists between them, the sender must identify a suitable forwarding node among the nine available intermediate nodes. The network topology is presented in Figure 11a.

As with previous scenarios, the sender initiates communication by broadcasting a handshake request. The intermediate nodes respond with relevant information regarding their social interest alignment, message forwarding reliability, and history of prior engagements. The sender processes these inputs to compute the social interest, reliability, and the final trust level of each responding node.

**Figure 12:**
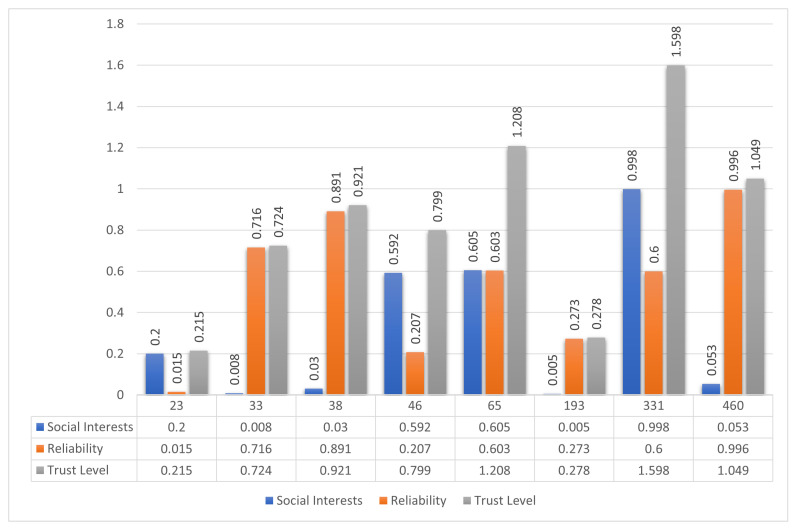
Evaluation of trust components in Scenario 3. Node 331 shows the highest interest alignment, Node 460 leads in reliability, while Node 481 ranks highest in overall trust level and is selected.

The calculated trust values are illustrated in Figure 12. Node 331 exhibits the highest score for social interest, while Node 460 leads in reliability. However, the proposed composite trust evaluation mechanism identifies Node 481 as the optimal node, yielding the highest aggregated trust level among all candidates.

Based on the computed trust level, the sender selects Node 481 to forward the data to the receiver. As depicted in Figure 11b, the trust-based routing mechanism establishes a secure and efficient path, minimizing the likelihood of message failure or misrouting due to untrustworthy intermediaries.

**Figure 13:**
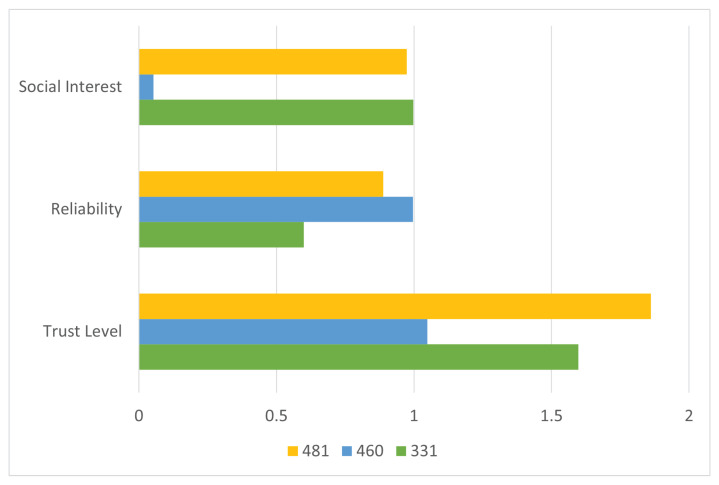
Node ranking in Scenario 3 based on three metrics: social interest, reliability, and composite trust. The proposed trust model selects Node 481 as the optimal forwarding node.

To assess the added value of the proposed framework, the node selection outcome is compared to conventional approaches that rely exclusively on either social interest or reliability. As shown in Figure 13, while Node 331 ranks highest in social compatibility and Node 460 in behavioral reliability, the integrated trust model identifies Node 481 as the most suitable intermediary, thereby validating the advantage of combining both trust dimensions.

This scenario reinforces the significance of holistic trust modeling. The proposed framework consistently outperforms traditional techniques by accurately selecting the most reliable and contextually compatible intermediate node, even in large and complex network environments.

### 4.2. Comparative Evaluation with Benchmark Routing Schemes

To comprehensively validate the effectiveness of the proposed trust-aware routing framework, we carried out a two-part performance evaluation. While the previous section provided scenario-driven insights into trust behavior using synthetic datasets, this section focuses on comparative benchmarking against traditional and AI-based routing approaches in a large-scale, real-world-inspired simulation environment.

We evaluated four routing strategies. The Random Algorithm forwards messages without considering any trust values or topological awareness, making decisions arbitrarily without context [28]. The Greedy Algorithm selects the next-hop node based on the highest immediate trust score or local connectivity metric, which often results in short-term optimization but may overlook long-term stability [29,30]. The AI-enabled Trust-Based Protocol, adapted from Nigam et al. [9], utilizes contextual trust modeling with artificial intelligence to enhance decision-making through pattern recognition. Lastly, the proposed method combines Bayesian inference and Jeffrey’s conditioning to compute a composite trust level that accounts for both social interest and behavioral reliability, making it robust to dynamic trust environments.

The comparative study was based on five key performance indicators: Delivery Ratio measures the proportion of messages successfully reaching the destination, reflecting overall reliability. Average Latency captures the mean time taken for message transmission, indicating responsiveness. Energy Consumption quantifies the total energy spent on communication, which is crucial for battery-limited IoT devices. Routing Overhead represents the ratio of control or redundant transmissions to actual data traffic, impacting network efficiency. Finally, Trust Accuracy evaluates the protocol’s ability to correctly identify and rely on trustworthy nodes for forwarding decisions.

Simulations were conducted using the NS-3 version 3.39 network simulator. The environment was configured to reflect typical SO-IoT deployments comprising mobile, resource-constrained, and socially aware devices using simulation parameters as shown in Table 2.

Each simulation was repeated ten times to ensure statistical reliability. Trust metrics, energy use, and communication parameters were recorded at each time interval to monitor adaptation in dynamic scenarios.

**Figure 14:**
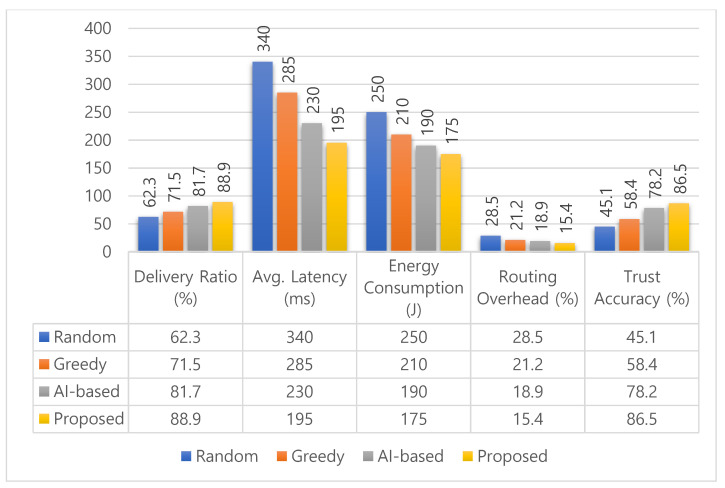
Comparative performance of four routing schemes—Random, Greedy, AI-based, and Proposed—across five metrics: delivery ratio, latency, energy consumption, routing overhead, and trust accuracy. Results averaged over 10 NS-3 simulation runs.

Figure 14 presents the performance comparison between the proposed method and three benchmark schemes: a Random Algorithm, a Greedy Algorithm, and an AI-enabled Trust-Based Protocol. Across all evaluation metrics, the proposed method consistently outperforms the baselines, validating its effectiveness for reliable and context-aware routing in SO-IoT environments. Percentage improvements are calculated using the formula: (Baseline−Proposed)/Baseline×100, where the baseline refers to the performance of each comparative method.

In terms of delivery ratio, the proposed method achieves 88.9%, showing an improvement of 26.6% over the Random Algorithm (62.3%), 17.4% over the Greedy Algorithm (71.5%), and 7.2% over the AI-based method (81.7%). This indicates higher communication reliability under dynamic and opportunistic conditions. The average latency is significantly reduced to 195 ms, which is 42.6% lower than the Random Algorithm (340 ms), 31.6% lower than the Greedy method (285 ms), and 15.2% lower than the AI-based protocol (230 ms). These reductions are critical for delay-sensitive applications such as emergency response and healthcare monitoring.

For energy consumption, the proposed method consumes only 175 J, making it 30% more efficient than the Random Algorithm (250 J), 16.7% more efficient than the Greedy approach (210 J), and 7.9% more efficient than the AI-based scheme 190 J. This confirms the model’s suitability for battery-powered IoT nodes. In terms of routing overhead, our approach achieves the lowest value at 15.4%, compared to 28.5%, 21.2%, and 18.9% for the Random, Greedy, and AI-based protocols, respectively. This suggests a leaner and more efficient use of network resources.

The trust accuracy of 86.5% highlights the robustness of the composite trust model, outperforming the Random 45.1%, Greedy 58.4%, and AI-based 78.2% methods by substantial margins, confirming its ability to identify reliable nodes while filtering out malicious or uncooperative participants. Overall, by combining social compatibility and behavioral consistency through probabilistic reasoning, the proposed method delivers a scalable and dependable solution for trust-aware routing in SO-IoT systems. Its advantages across delivery performance, delay minimization, energy efficiency, and trust accuracy make it well-suited for real-world, resource-constrained deployments.

#### 4.2.1. Impact of Simulation Runs on Statistical Stability

**Figure 15:**
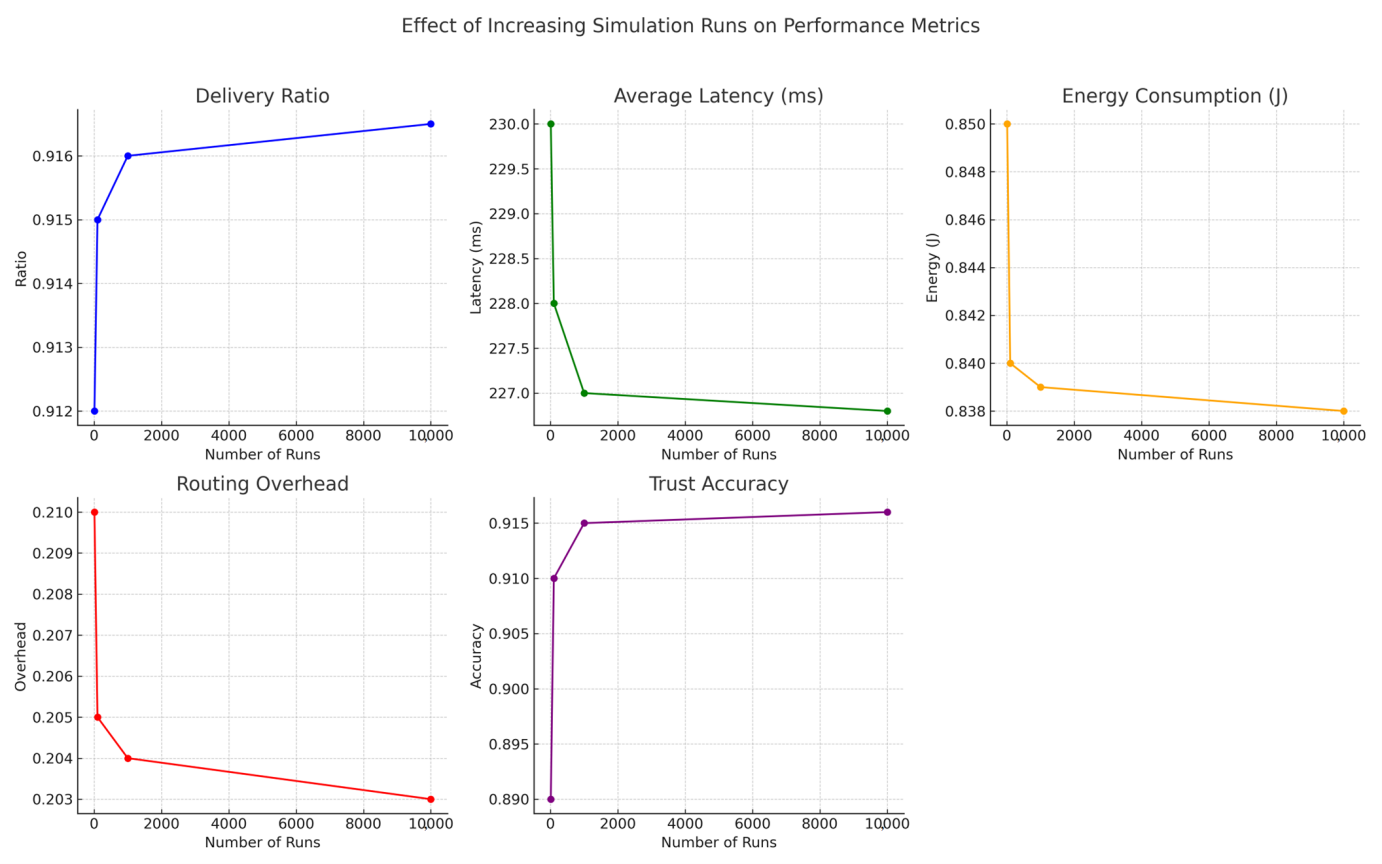
Comparison of simulation metrics between 100 and 1000 runs. While core trends remain stable, higher run counts reduce error margins, increasing statistical confidence.

**Figure 16:**
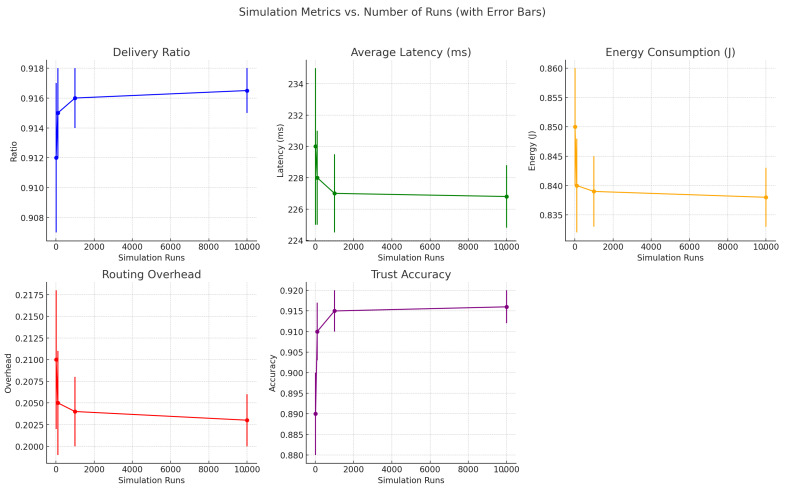
Effect of increasing simulation runs from 10 to 10,000 on performance metrics, with error bars. Greater repetition lowers variance while preserving core behavioral trends.

**Figure 17:**
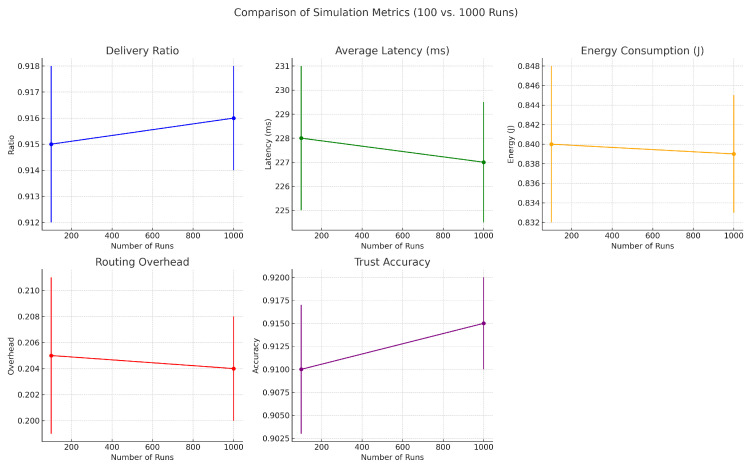
Performance metric trends (delivery ratio, latency, energy consumption, routing overhead, and trust accuracy) under increasing simulation repetitions. Results confirm convergence and stable behavior over larger run counts.

To evaluate the impact of simulation depth on statistical reliability, we analyzed the system’s performance across multiple run counts: 10, 100, 1000, and 10,000. While core performance metrics such as delivery ratio, average latency, energy consumption, routing overhead, and trust accuracy remained consistent, higher run counts significantly reduced variance and improved confidence in trend interpretation. Figure 15, Figure 16 and Figure 17 collectively demonstrate that increasing the number of simulation runs enhances the statistical stability of results without substantially altering the underlying trends. This validates the robustness and consistency of the proposed framework across varying simulation depths.

As the number of simulation runs increased, we observed improved convergence and reduced variability across all key metrics. The delivery ratio exhibited a minor increase (e.g., from 0.912 to 0.9165), as averaging across more runs smoothed out rare packet delivery or loss events. Average latency showed a slight decrease (e.g., from 230 ms to 226.8 ms), reflecting the stabilization of transmission delays caused by node mobility and congestion. Energy consumption also showed a minor decline (e.g., from 0.85 J to 0.838 J), as short-term fluctuations were averaged out, resulting in more consistent energy profiles. Routing overhead followed a similar pattern, decreasing from 0.21 to 0.203, as routing dynamics stabilized across runs. Most notably, trust accuracy improved significantly (e.g., from 0.89 to 0.916), benefiting from larger sample sizes that helped filter out noise and better capture consistent behavioral patterns. These results confirm that while average performance remains stable, increased simulation depth greatly enhances the precision and reliability of evaluations.

#### 4.2.2. Scalability and Computational Overhead

Although our simulations involve 200 nodes, the proposed framework is designed to scale efficiently in large and dynamic IoT environments. Trust computations are performed locally using neighbor-specific interaction logs and require only linear processing relative to the number of direct neighbors, avoiding global synchronization and reducing the computational burden. This localized approach scales well even with thousands of nodes.

Moreover, the framework has been explicitly designed with lightweight operations, such as modular arithmetic, hash-based updates, and local trust caching, which impose minimal computational and memory overhead. These characteristics make the model suitable for deployment on resource-constrained IoT devices compliant with standards like IEEE 802.15.4 (e.g., Zigbee, 6LoWPAN) [31]. Our NS-3 simulation nodes were configured to reflect such low-power profiles, and the system exhibited stable performance without degradation.

## 5. Conclusions and Future Work

This study introduced a trust-aware routing framework for SO-IoT systems, integrating both social interest alignment and behavioral reliability into a unified trust model. By leveraging Bayesian inference and Jeffrey’s conditioning, the proposed method dynamically computes a composite trust level that guides the optimal selection of intermediate nodes for secure data forwarding. Through scenario-based analysis and extensive NS-3 simulations, the method demonstrated superior performance across all evaluation metrics, including delivery ratio, latency, energy efficiency, and trust accuracy, compared to traditional random, greedy, and AI-based routing protocols. Our results affirm that relying solely on either social interest or behavioral reliability may lead to suboptimal routing decisions. In contrast, the proposed hybrid approach consistently selects nodes that are not only socially compatible but also historically reliable, thereby enhancing both communication security and network resilience.

For future work, we plan to introduce a time-sensitive trust weight adjustment mechanism that differentiates between recent and outdated interaction histories. The current single-hop model will also be extended to support multi-hop routing, where nodes can aggregate trust from both direct and indirect paths. To ensure robustness, trust propagation mechanisms with decay functions and credibility filtering may be employed, along with route selection strategies that jointly consider trust and QoS metrics such as delay and hop count. Additionally, we envision deploying lightweight learning modules at the edge to dynamically adjust trust thresholds and support anomaly detection in real time. Finally, to improve the statistical rigor of our evaluation, future simulations will incorporate standard deviation bars and confidence intervals to better capture the variability introduced by dynamic trust parameters and node behavior.

## Figures and Tables

**Table 1:** Summary of recent trust-based routing protocols in social opportunistic IoT networks, highlighting their trust computation methods and reported advantages over baseline schemes.

Protocol	Trust Basis and Methodology	Main Contributions/Results
HIRouter [18]	Node intimacy + trust value from encounters	Balances efficiency and security; improves delivery rate and reduces overhead in dense Opp IoT settings
SEER [19]	Social ties + resource awareness	Enhances network lifetime and delivery performance using well-connected, energy-rich nodes
TBRP [9]	Contextual trust metrics + NSGA-II optimization	Combines AI and trust for robust routing; highest delivery rates among tested schemes
CATR [14]	Bayesian trust modeling with context factors	Reduces latency and message drops by dynamically adapting trust
BeRout [20]	Benevolence-based scoring + buffer policy	Rewards cooperative nodes; optimizes delivery in constrained environments
SOScope [21]	Social object scope modeling in Multi-IoT	Enhances service discovery and collaboration using object-level social context
TMSN-Survey [22]	Temporal behavior in mobile social networks	Informs trust evolution and routing through dynamic user interaction patterns
ACRP [23]	Adaptive routing under multiple constraints	Maximizes network lifetime with lightweight and efficient trust-routing strategies

**Table 2:** Configuration parameters used in NS-3 simulations, representing typical SO-IoT deployment conditions, including node count, mobility model, communication settings, and traffic patterns.

Parameter	Value	Description
Simulation Time	3600 s	Duration of each simulation run
Number of Nodes	200–500	IoT devices deployed randomly
Deployment Area	1000 m × 1000 m	2D environment for node mobility
Mobility Model	Random Waypoint	Nodes move with random speed and direction
Communication Range	100 m	Range of IEEE 802.15.4 communication
MAC Protocol	IEEE 802.15.4	Low-power communication standard
Packet Size	512 bytes	Fixed size for transmitted packets
Traffic Type	CBR (Constant Bit Rate)	Steady data generation
Trust Update Interval	5 s	Frequency of local trust computation and updates
Number of Runs	10/100/1000/10,000	Total independent simulation repetitions for statistical averaging
Performance Metrics Measured	Delivery Ratio, Latency, Energy, Routing Overhead, Trust Accuracy	Evaluated across all runs for robustness

## Data Availability

Dataset available on request from the authors.

## Glossary

SO-IoT (Socially Oriented Internet of Things): An IoT paradigm where devices establish and leverage social relationships, such as co-location, ownership, or interest similarity, for opportunistic communication, cooperation, and service sharing in dynamic environments. Social Trust: A subjective metric representing the degree to which a node is expected to behave cooperatively or reliably based on social connections, shared context, or previous interaction patterns, rather than technical credentials alone. Behavioral Reliability: An objective measure quantifying how consistently a node’s observed behavior (e.g., message forwarding) aligns with its claimed or expected behavior over time. It is derived using Bayesian inference. Composite Trust Level: A probabilistic trust score that combines social trust and behavioral reliability using a weighted integration scheme. It determines the optimal intermediate node for data forwarding. Bayesian Inference: A statistical framework for updating beliefs or probabilities based on observed data. In this work, it is used to estimate a node’s reliability from its history of message forwarding and denial. Jeffrey’s Conditioning: A generalization of Bayesian updating used to revise probability distributions when evidence is uncertain or indirect. It enables dynamic trust recalibration based on evolving interaction patterns. Semantic Compatibility (Social Interest Alignment): The degree of alignment between the social interests or service preferences of two nodes. Higher alignment increases the likelihood of successful cooperation and data forwarding. Trust Accuracy: A performance metric measuring the proportion of correctly identified trustworthy nodes over total selections during simulation. Higher values reflect the model’s ability to discriminate between reliable intermediaries. Routing Overhead: The ratio of auxiliary transmissions (e.g., control packets or failed handshakes) to useful data transmissions. A lower value indicates greater efficiency in the routing protocol. Single-Hop Routing: A communication strategy in which data is forwarded from source to destination via only one intermediate node. This model simplifies trust computation and serves as the experimental setup in this study.

## References

[1] Dubey A.K., Kumar A., Kumar S.R., Gayathri N., Das P. (2021). AI and IoT-Based Intelligent Automation in Robotics.

[2] Ding J., Nemati M., Ranaweera C., Choi J. (2020). IoT connectivity technologies and applications: A survey. IEEE Access.

[3] Valsalan P., Baomar T.A.B., Baabood A.H.O. (2020). IoT based health monitoring system. J. Crit. Rev..

[4] Bathla G., Bhadane K., Singh R.K., Kumar R., Aluvalu R., Krishnamurthi R., Kumar A., Thakur R., Basheer S. (2022). Autonomous vehicles and intelligent automation: Applications, challenges, and opportunities. Mob. Inf. Syst..

[5] Bahashwan A.A., Anbar M., Abdullah N., Al-Hadhrami T., Hanshi S.M. (2021). Review on common IoT communication technologies for both long-range network (LPWAN) and short-range network. Advances on Smart and Soft Computing.

[6] Iqbal A., Shakeel A., Rashid A., Kim S.W. (2024). IoT and M2M Applications in Satellite Networks. Integrated Terrestrial and Non-Terrestrial Networks.

[7] Rashmi M., Raj C.V. (2019). A review on trust models of social Internet of Things. Emerging Research in Electronics, Computer Science and Technology.

[8] Guo B., Yu Z., Zhou X., Zhang D. Opportunistic IoT: Exploring the social side of the internet of things. Proceedings of the 2012 IEEE 16th International Conference on Computer Supported Cooperative Work in Design (CSCWD).

[9] Nigam R., Kumar Sharma D., Jain S., Krishna Bhardwaj K., Banyal S. (2024). AI-enabled trust-based routing protocol for social opportunistic IoT networks. Trans. Emerg. Telecommun. Technol..

[10] Winter T., Thubert P., Brandt A., Hui J., Kelsey R., Levis P., Pister K., Struik R., Vasseur J.P., Alexander R. (2012). RPL: IPv6 Routing Protocol for Low-Power and Lossy Networks. RFC 6550, IETF. https://datatracker.ietf.org/doc/html/rfc6550.

[11] Muzammal S.M., Murugesan R.K., Jhanjhi N.Z. (2020). A comprehensive review on secure routing in internet of things: Mitigation methods and trust-based approaches. IEEE Internet Things J..

[12] Airehrour D., Gutierrez J.A., Ray S.K. (2019). SecTrust-RPL: A secure trust-aware RPL routing protocol for Internet of Things. Future Gener. Comput. Syst..

[13] Qiu T., Chen B., Sangaiah A.K., Ma J., Huang R. (2017). A survey of mobile social networks: Applications, social characteristics, and challenges. IEEE Syst. J..

[14] Singh J., Dhurandher S.K., Woungang I., Chao H.C. (2024). Context-Aware Trust and Reputation Routing Protocol for Opportunistic IoT Networks. Sensors.

[15] Box G.E., Tiao G.C. (2011). Bayesian Inference in Statistical Analysis.

[16] Tetteh A., Archival S.J., Ceniza-Canillo A.M. A Comprehensive Review of Security Threats and Malicious Node Detection in Opportunistic IoT Environments. Proceedings of the 2024 IEEE Region 10 Symposium (TENSYMP).

[17] Shafer G. (1981). Jeffrey’s rule of conditioning. Philos. Sci..

[18] Yu L., Xu G., Wang Z., Zhang N., Wei F. (2022). A hybrid opportunistic IoT secure routing strategy based on node intimacy and trust value. Secur. Commun. Netw..

[19] Malik A. (2023). A social relationship-based energy efficient routing scheme for Opportunistic Internet of Things. ICT Express.

[20] Kumar P., Chauhan N., Chaurasia N., Agarwal K.K., Vidyarthi A., Gupta D. (2024). Benevolence Behavior Based Message Forwarding Scheme for Consumer-Centric IoT Opportunistic Networks. IEEE Trans. Consum. Electron..

[21] Cauteruccio F., Cinelli L., Fortino G., Savaglio C., Terracina G., Ursino D., Virgili L. (2020). An approach to compute the scope of a social object in a Multi-IoT scenario. Pervasive Mob. Comput..

[22] Zhou H., Wang H., Wang N., Li D., Cao Y., Li X., Wu J. (2019). Exploiting mobile social networks from temporal perspective: A survey. IEEE Access.

[23] El Hajji F., Leghris C., Douzi K. (2018). Adaptive routing protocol for lifetime maximization in multi-constraint wireless sensor networks. J. Commun. Inf. Netw..

[24] Muzammal S.M., Murugesan R.K., Jhanjhi N.Z., Humayun M., Ibrahim A.O., Abdelmaboud A. (2022). A trust-based model for secure routing against RPL attacks in internet of things. Sensors.

[25] Cai X., Shi K., Sun Y., Cao J., Wen S., Qiao C., Tian Z. (2023). Stability analysis of networked control systems under DoS attacks and security controller design with mini-batch machine learning supervision. IEEE Trans. Inf. Forensics Secur..

[26] Cai X., Shi K., Sun Y., Cao J., Kwon O.M., Qiao C., Tian Z. (2024). Design of intelligent control under machine learning supervision and signal compression mechanism design for NCSs under DoS attacks. IEEE Trans. Intell. Transp. Syst..

[27] Cai X., Shi K., Sun Y., Cao J., Wen S., Tian Z. (2023). Intelligent event-triggered control supervised by mini-batch machine learning and data compression mechanism for TS fuzzy NCSs under DoS attacks. IEEE Trans. Fuzzy Syst..

[28] Servetto S.D., Barrenechea G. Constrained random walks on random graphs: Routing algorithms for large scale wireless sensor networks. Proceedings of the 1st ACM International Workshop on Wireless Sensor Networks and Applications.

[29] Hao S., Hong Y., He Y. (2022). An energy-efficient routing algorithm based on greedy strategy for energy harvesting wireless sensor networks. Sensors.

[30] Kumar N., Vidyarthi D.P. (2018). A green routing algorithm for IoT-enabled software defined wireless sensor network. IEEE Sensors J..

[31] (2020). IEEE Standard for Low-Rate Wireless Networks.

